# Cementless Ceramic-on-Ceramic Total Hip Replacement in Children and Adolescents

**DOI:** 10.3390/children8100858

**Published:** 2021-09-27

**Authors:** Giovanni Trisolino, Stefano Stallone, Francesco Castagnini, Barbara Bordini, Monica Cosentino, Stefano Lucchini, Paola Zarantonello, Daniele Ferrari, Dante Dallari, Francesco Traina

**Affiliations:** 1 Pediatric Orthopedics and Traumatology, IRCCS Istituto Ortopedico Rizzoli, 40136 Bologna, Italy; paola.zarantonello@ior.it (P.Z.); daniele.ferrari@ior.it (D.F.); 2 Orthopaedic-Traumatology and Prosthetic Surgery and Revisions of Hip and Knee, IRCCS Istituto Ortopedico Rizzoli, 40136 Bologna, Italy; francesco.castagnini@ior.it (F.C.); stefano.lucchini@ior.it (S.L.); francesco.traina@ior.it (F.T.); 3 Medical Technology Laboratory, IRCCS Istituto Ortopedico Rizzoli, 40136 Bologna, Italy; barbara.bordini@ior.it (B.B.); monica.cosentino@ior.it (M.C.); 4 Conservative Orthopedic Surgery and Innovative Techniques, IRCCS Istituto Ortopedico Rizzoli, 40136 Bologna, Italy; dante.dallari@ior.it

**Keywords:** arthroplasty, replacement, hip, child, adolescent, ceramics, cementless, sport

## Abstract

*Background*: total hip replacement (THR) is a rare surgical option in children and adolescents with disabling hip diseases. The aim of this study is to report results from a retrospective cohort of patients aged 18 years or less who underwent cementless Ceramic-on-Ceramic (CoC) THR at a single institution, investigating clinical and radiographic outcomes, survival rates, and reasons for revision of the implants. *Materials and methods*: we queried the Registry of Prosthetic Orthopedic Implants (RIPO) to identify all children and adolescents undergoing THR between 2000 and 2019 at a single Institution. Inclusion criteria were patients undergoing cementless CoC THR, aged less than 18 years at surgery, followed for at least 2 years. Sixty-eight patients (74 hips) matched all the inclusion criteria and were enrolled in the study. We assessed the clinical and radiographic outcomes, the rate of complications, the survival rate, and reasons for revision of the implants. *Results*: The mean follow-up was 6.6 ± 4.4 years (range 2–20). The most frequent reason for THR was post-traumatic or chemotherapy-induced avascular necrosis (38%). The overall survival rate of the cohort was 97.6% (95% CI: 84.9–99.7%) at 5 years of follow-up, 94.4% (95% CI: 79.8–98.6%) at 10 years and 15 years of follow-up. Two THR in two patients (2.7%) required revision. With the numbers available, Cox regression analysis could not detect any significant interaction between preoperative or intraoperative variables and implant survivorship (*p*-value 0.242 to 0.989).” The average HOOS was 85 ± 14.3 (range 30.6–100). Overall, 23 patients (48%) reported excellent HOOS scores (>90 points), 21 patients (44%) reported acceptable HOOS scores (60–90 points) while 4 patients (8%) reported poor outcomes (<60 points). Twenty-one patients (43%) were regularly involved into moderate- to high-intensity sport activities (UCLA ≥ 6). *Conclusions*: Cementless CoC THR is a successful procedure in children and teenagers, having demonstrated high implant survivorship and low rates of complications and failure. A meticulous preoperative planning and implant selection is mandatory, to avoid implant malposition, which is the main reason of failure and revision in these cases. Further studies are needed to assess the impact of the THR on the psychosocial wellbeing of teenagers, as well as risks and benefits and cost-effectiveness in comparison to the hip preserving surgical procedures.

## 1. Introduction

Disabling hip diseases in children and adolescents may be due to a wide number of congenital or developmental pathologies, such as avascular necrosis (AVN) Legg-Calvè-Perthes disease (LCPD), slipped capital femoral epiphysis (SCFE), juvenile idiopathic arthritis (JIA), developmental dysplasia of the hip (DDH) [1]. Despite a plethora of “hip preserving” surgical procedures have been developed in recent decades, joint salvage may be an unsatisfying solution in the case of end-stage degenerative hip disease, with severe pain, disability and overall decreased quality of life [2,3,4].

Total hip replacement (THR) is a safe and effective option for treating end-stage hip osteoarthritis in adults, but concerns arise when this solution is considered for children and adolescents. Historically, skeletal immaturity, anatomic abnormalities, and active life expectancy have been powerful deterrents leading to technical pitfalls, bearing wear, premature loosening, and possible multiple revisions [5,6,7,8].

To date, the current literature reported 10–15% 10-year revision rate in very young patients, regardless the bearing, apparently higher than in adults [9,10]. However, the few studies about pediatric THR are generally small case series with limited follow-ups, sometimes including children treated for malignancies and obsolete implants/bearings [11,12,13].

During recent decades, cementless THR with hard-on-hard bearing surfaces, such as ceramic-on-ceramic (CoC) bearings, has emerged as a suitable option in young patients, having demonstrated superior tribological properties, with minimum wear and osteolysis [14,15]. These features may make CoC potentially ideal for children and adolescents. Nonetheless, concerns have been raised about brittleness, and possible bearing fracture, potentially leading to early, premature revisions [16]. So far, long-term results from sizeable case series are universally sought [9,10,17].

Therefore, we evaluated a consecutive cohort of patients, aged 18 years or younger, who underwent cementless CoC THR for non-oncological reasons in a tertiary center. The purposes of the present study were the evaluation of: (1) the clinical and radiographic outcomes; (2) the rate of complications, the survival rate, and reasons for revision of the implants.

## 2. Materials and Methods

### 2.1. Study Design and Population

This is a retrospective analysis of prospectively collected data. We queried the Registry of Prosthetic Orthopedic Implants (RIPO) to identify all children and adolescents undergoing THR between 2000 and 2019 at a single Institution. RIPO is the Emilia-Romagna regional arthroplasty registry. It includes 68 Orthopedic facilities in 59 public and private hospitals, involving 4,450,000 inhabitants. RIPO has been actively collecting and following primary joint (hip, knee and shoulder) replacement procedures and revision surgeries, since 2000. Standard forms are used to capture data about demographics, preoperative diagnosis, fixation and type (batch and code) of the implants. The capture rate of RIPO is 98%, the lack of adhesion being responsible for the missing data (2%).

Inclusion criteria were patients undergoing cementless CoC THR, treated at a single tertiary referral center, aged less than 18 years at surgery, followed for at least 2 years. Patients were excluded in the case of resection endoprosthesis for malignancies and other oncological reasons.

### 2.2. Patients Evaluation

Demographics and clinical variables at baseline, including age, sex, weight, height, BMI, and related z-scores, based on the Italian reference charts [18], laterality, reasons for THR, were explored by medical charts.

Surgical data included type of anesthesia, surgical approach, type of implant, intraoperative, and postoperative complications, implant failure, and reasons for revision.

Patients were contacted by mail or by phone and were asked to fulfill the Italian version of the Hip disability and Osteoarthritis Outcome Score (HOOS) [19], the University of California at Los Angeles (UCLA) Activity Score [20] and EQ-5D [21] questionnaire. The UCLA activity score is a ten-point activity scale that evaluates patient activity based on 10 descriptive activity levels ranging from wholly inactive (level 1) to regular participation in impact sports (level 10). The EQ-5D provides a simple descriptive profile and a single summary index value for health status that can be used in evaluation of health care. The EQ-5D-3L comprises five dimensions, each describing a different aspect of health: mobility, self-care, usual activities, pain /discomfort and anxiety/depression. Concerning the HOOS, reference values for young adult population (age 18–34 years) were obtained from literature, since we lack preoperative scores in most of the patients. According to these values, outcomes were considered excellent for HOOS > 90 points, acceptable for 60 ≤ HOOS ≤ 90 points, and poor for HOOS < 60 points, in each domain [22].

Plain radiographs were evaluated preoperatively and at the most recent follow-up, after appropriate calibration. Positional parameters were assessed according to values guidelines from published literature, as listed in Table 1 [23,24,25,26,27,28,29,30,31]. Femoral stem and acetabular cup osteointegration were quantified according to Engh [32] and Moore scale [33]. Heterotopic ossifications were graded according to the Brooker system [34].

### 2.3. Statistical Analysis

Continuous data were expressed as means, whereas categorical and ordinal data were expressed as absolute values and percentages. Demographics, implant-related features, reasons for causes of revision were reported as raw data, ranges, and percentages. The survival curves were calculated and plotted according to Kaplan-Meier method. The curve starts by definition at 100% survival the moment when the period of follow-up begins. The implant is considered to be ‘surviving’ up to when it was necessary to replace even a single component. A 95% confidence interval was calculated. The survival times of unrevised implants were considered at the last date of observation (date of death or July 31, 2021, for pediatric patients). Statistical analyses were performed using SPSS software (version 14.0.1, Chicago, IL, USA) and JMP, version 12.0.1 (SAS Institute Inc., Cary, NC, USA, 1989–2007).

## 3. Results

### 3.1. Patients’ Population

On a total of 134,000 patients treated with THR in our region, the RIPO counted a total of 129 hips (0.09%) in 122 patients aged 18 years or less. Ninety-nine hips (96 patients) were treated at our Institution. Of them, 17 procedures were excluded because they underwent THR for malignancies and 8 were excluded because of bearings others than CoC. Sixty-four hips (68 patients) matched all the inclusion criteria and were enrolled in the study. Demographics and preoperative clinical features are summarized in Table 2. Noticeably, 8 patients (11.8%) had short stature (<3rd percentile) and 11 patients (16.2%) were obese (BMI > 95th percentile).

**Figure 1:**
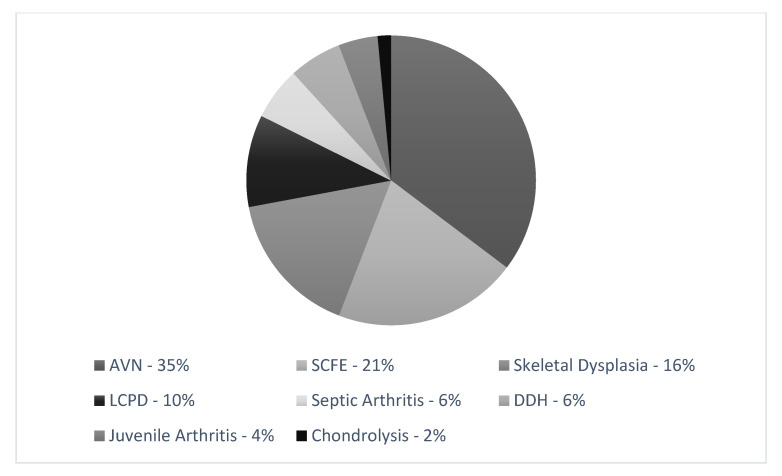
Causes of THR per patient. AVN: avascular necrosis; SCFE: slipped capital femoral epiphysis; LCPD: Legg-Calvè-Perthes disease; DDH: developmental dysplasia of the hip.

The most frequent reason for THR was post-traumatic or chemotherapy-induced AVN (38%). Forty-two patients (43 hips, 58.1%) had at least one previous hip operation, mostly after fracture, SCFE, and core decompression. Twelve patients (15.7%) were diagnosed with genetic skeletal dysplasia (Figure 1).

### 3.2. Surgical Data

The surgical approach was lateral in 54 cases (73%), anterior in 16 cases (21.6%), postero-lateral in 4 cases (5.4%). The most implanted THR was FIXA-TiPor^®^ (Adler-Ortho, Milan-Italy) a highly porous titanium cup (44 implants 59.5%) [10]. The smallest implant size was used in 11 cases (14.9%). The stem design was anatomical in 25 hips (33.8%), rectangular in 17 hips (23%), conical in 13 hips (17.6%), and a mini stem was used in 19 hips (25.7%). Forty implants (54%) had a fully coated stem. A modular neck was used in 27 hips (36.5%). Regarding the bearing surface, the BIOLOX^®^delta ceramic was used in 60 cases (81.1%), while 14 hips (18.9%) received Biolox or Biolox-Forte bearings (CeramTec, Plochingen-Germany) (Table 3).

Perioperative complications included: intraoperative femoral fracture requiring cerclages in 2 cases (2.7%). Postoperative bleeding required blood transfusion in 48 cases (64.9%: 30 autologous, 18 homologous) and embolization of the medial circumflex artery in one case (1.3%).

**Figure 2:**
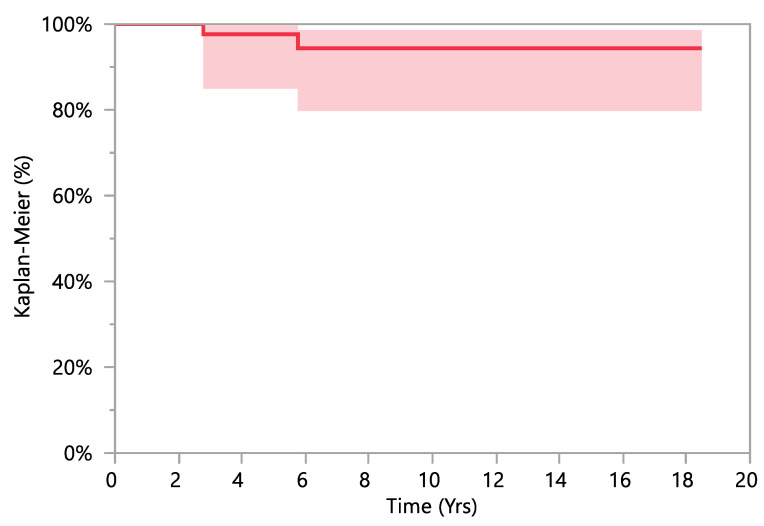
Survivorship of THR implanted in our cohort. The survival curves were calculated and plotted according to Kaplan-Meier method. The overall survival rate of the cohort was 97.6% at 5 years of follow-up, 94.4% at 10 years and 15 years of follow-up.

The mean follow-up was 6.6 ± 4.4 years (range 2–20). Two THR in two patients required revision. One patient with Albers–Schömberg disease required stem revision 33 months after the index procedure, due to stem undersizing and subsidence. Another patient with CDH sequelae underwent cup revision 68 months after the index procedure, because of recurrent hip instability due to initial vertical malposition of the cup. The overall survival rate of the cohort was 97.6% (95% CI: 84.9–99.7%) at 5 years of follow-up, 94.4% (95% CI: 79.8–98.6%) at 10 years and 15 years of follow-up (Figure 2). With the available data, Cox regression analysis could not detect any significant interaction between preoperative or intraoperative variables and the survivorship (*p*-value 0.242 to 0.989).

### 3.3. Clinical Evaluation

**Figure 3:**
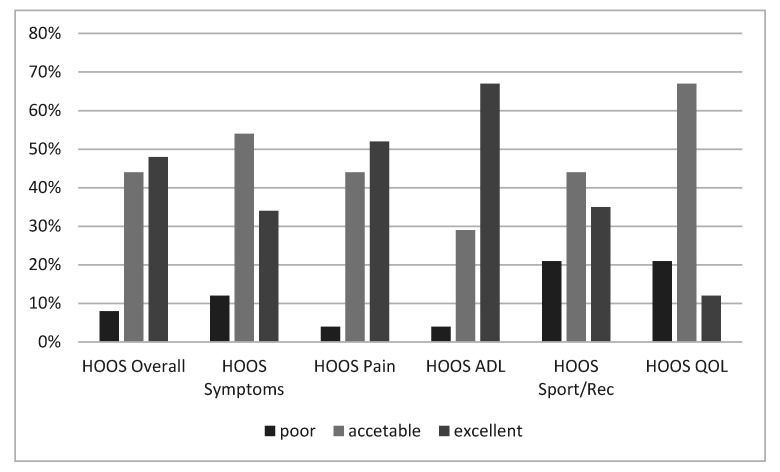
HOOS score. Outcomes were considered excellent for HOOS > 90 points, acceptable for 60 ≤ HOOS ≤ 90 points, and poor for HOOS < 60 points, in each domain.

Forty-eight of the original 68 children (71%) were available for subjective clinical evaluation. Among the non-participants, six patients were definitely unreachable by phone, e-mail, or letter, while 14 patients refused to complete the questionnaires, although they did not refer any problem related to the THR. The average HOOS was 85 ± 14.3 (range 30.6–100). Overall, 23 patients (47.9%) reported excellent HOOS scores, 21 patients (43.8%) reported acceptable HOOS scores, while 4 patients (8.3%) reported poor outcomes (Figure 3). Among those patients who reported poor outcomes, one patient underwent cup revision, one patient was successfully implanted but she is still waiting for contralateral THR, one patient had bilateral THR for chemotherapy-induced AVN, and one patient had poor outcomes despite the THR was apparently well implanted and did not show any aspect of failure at the most recent radiographs.

**Figure 4:**
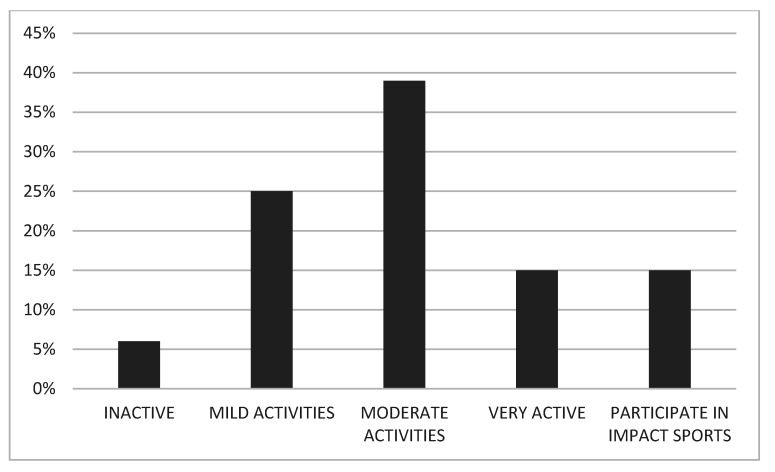
UCLA Activity Score results. Outcomes were grouped in: 0–2: inactive; 3–4: mild activities; 5–6: moderate activities; 7–8: very active; 9–10: participate in impact sports.

Twenty-one patients (43.8%) were regularly involved into moderate- to high-intensity sport activities (UCLA ≥ 6) (Figure 4). Seven of them (14.6%) performed high impact sport activities, such as skiing, tennis, and gym.

The mean EQ-5D 3L was 0.8 (range 0.045–1). Sixteen patients (33.3%) reported the maximum score, while two patients (4%) reported a score lower than 0.5. Both these patients underwent several previous surgeries, for treating DDH sequelae in one case, and JIA in the other case. 

### 3.4. Radiographic Evaluation

Preoperative and postoperative radiographic data are reported in Table 4. Preoperatively, 18 THR (24.3%) were implanted in skeletally immature children with closed triradiate cartilage (1 ≤ Risser ≤ 3). Radiographic acetabular insufficiency (LCEA < 25° and AI > 13°) was detected in 14 hips (18.9%), while protrusion was present in 3 hips (4.1%) An excessive valgus of the femoral neck was observed in 19 hips (25.7%), while an important varus deformity was present in 7 hips (9.5%).

Postoperative radiographs showed a significant vertical malposition of the cup in 1 case (1.4%), a significant varus stem in 8 cases (10.8%) and a significant valgus stem in 1 case (1.4%). Two patients (2.7%) showed moderate heterotopic bone formation (Brooker ≥ 2), that did not significantly affect the hip motion and symptoms. All those THR that did not undergo revision showed good radiographic osteointegration, with no evidence of implant breakage, radiographic lucencies, bone defects, cup migration, or stem subsidence at the most recent radiographs.

## 4. Discussion

THR is rarely required in children and teenagers. In our region, during the latest 20 years, less than 1‰ of THR were implanted in people less than 18 years old. This leads to a paucity of information regarding indication, timing, and prognosis of THR in very young individuals. 

Traditionally, the choice of THR in children has been usually postponed, for fear of early implant failure, especially in children with incomplete skeletal maturity. Nonetheless, in our cohort, the survival rate was very high, similar to the adult counterpart [35]. Moreover, the implant survival was satisfactory, regardless the degree of skeletal maturity, although we would like to point out that no THR was implanted in children with still open triradiate cartilage. Our findings are consistent with the current data from other national registries [9,10,36] and with some recent reports focused on THR implanted in very young people [11,17,37]. All these experiences support the impression that with the modern THRs, implant survival is no longer a concern in children and adolescents. In particular, cementless THRs with CoC coupling have shown excellent long-term survival rate in several studies [15,38,39], making this option most suitable in children and adolescents. Moreover, the advent of new generation ceramics, such as BIOLO*X*^®^delta allows use of large femoral heads, increasing the hip stability, and, likely, performance and duration of the THR.

In our experience, implant malposition was the only reason for implant revision. In one case, progressive subsidence was observed in a varus undersized stem, implanted in a boy with Albers–Schömberg disease; in another case, an excessively vertical cup with a 28 mm femoral head caused hip instability and required cup revision. 

THR can be a challenging procedure in very young people. The combination of distorted anatomy, small physique, and poor bone stock can impede proper implant placement. Implant sizing may be an issue in these patients. The combination of young age and peculiar conditions such as skeletal dysplasia may require particular attention in preoperative planning and implant selection. Noticeably, we used the smallest size of the cup in 11 hips (15%) and the smallest size of the stem in 14 hips (19%). A meticulous preoperative planning, even using simulation software, [40,41,42] and careful implant selection, sometimes requiring even customized implants [37], is crucial in such conditions, to prevent unpleasant pitfalls during the operation.

Clinical and functional outcomes and overall quality of life in children and adolescents with THR is another matter of concern. Despite the success of the operation, both in terms of implant stability and survival, only 48% of patients achieved overall excellent HOOS scores. Although pain was generally absent or mild after THR, symptoms such as residual stiffness and reduced hip motion could affect the performance of daily and high-demand sports activities, with obvious consequences on the patient’s quality of life. In our cohort only 43% of patients participated in moderate- to high-impact sports activities, and only 12% of patients reported an excellent quality of life, regardless of any preoperative parameter or intraoperative variable. The psychosocial implications of THR in such young individuals, even accounting for the cause of the THR, must be further investigated, to prepare the most appropriate therapeutic interventions, including physical therapy, patient education, and expectation management.

Major limitations to this study include the sample size, the retrospective design and the heterogeneity of the cohort in terms of initial diagnosis and previous procedures that may confound the long-term outcome. Moreover, almost 30% of patients did not complete the functional questionnaires.

## 5. Conclusions

Cementless CoC THR is a successful procedure in children and teenagers, having demonstrated high implant survivorship and low rates of complications and failure. A meticulous preoperative planning and implant selection is mandatory, to avoid implant malposition, which is the main reason of failure and revision in these cases. Further studies are needed to assess the impact of the THR on the psychosocial wellbeing of teenagers, as well as risks and benefits and cost-effectiveness, in comparison to the hip preserving surgical procedures.

## Figures and Tables

**Table 1:** Radiographic parameters.

*Radiographic Value*	*Description*	*Normal Value*	*Image*
**PREOPERATIVE**			
Lateral center-edge angle (lcea)	The angle measured between two lines drawn from the center of the best fit circle for the inferior and medial margins femoral head, one running vertically along the longitudinal axis of the pelvis and the other to the lateral acetabular rim [23,24]	25–39°	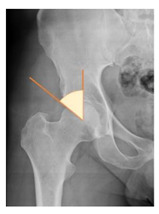
Acetabular index	The angle described between the line running through the medial edge of the sclerotic acetabular zone and through the lateral sourcil, and its horizontal line	3–13°	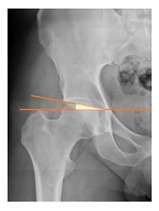
Acetabular depth/width ratio	The width is a line connecting the superolateral edge and the inferomedial edge of the acetabulum. The perpendicular line from the latter to the deepest part of acetabulum is the depth. The ADWR is the percentage of depth to the width [25]	>38%	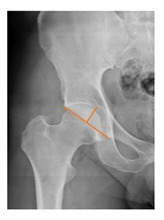
Sharp angle	the angle between a line passing from the superior to the inferior acetabular rim and the horizontal plane	<42°	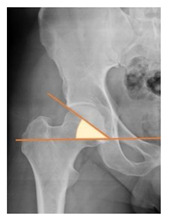
Neck-shaft angle	The angle between the longitudinal axes of the femoral neck and shaft	130 ± 5°	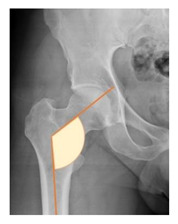
Risser	Measured according to “Risser +” grade [26]	0 to 5	
**POSTOPERATIVE**			
Cup abduction	The angle described between the interteardrop line and the tangent to the opening of the cup	35–45°	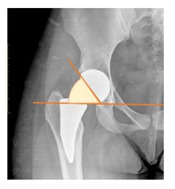
Cup anteversion	measured according to the Lewinnek’s [27] method	5–25°	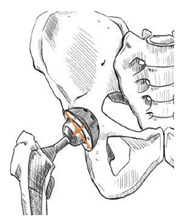
Center of rotation high	the perpendicular distance between the center or rotation and the interteradrop line		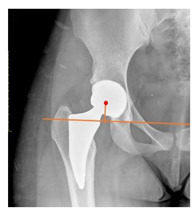
Center of rotation medialization	the perpendicular distance between the center of rotation and a line perpendicular to the interteardrop line and tangent to the medial border of the teardrop		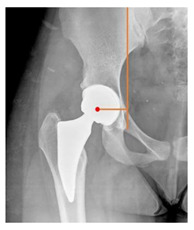
Stem varus-valgus	the angle between the axis of the femur and the main axis of the stem [28] +/−: the stem is valgus/varus	−5–+5°	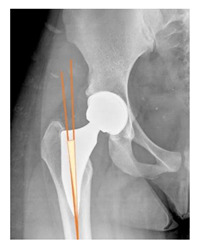
Leg length discrepancy	Trochanteric method: the difference between the distances from the tip of the lesser trochanter to the interteardrop line, measured in both the hips [29] +/−: the treated hip was longer/shorter	0 ± 10 mm	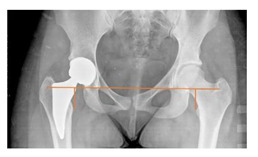
Femoral offset	the perpendicular distance between the center of rotation and the axis of femoral shaft [30]	41–44 mm	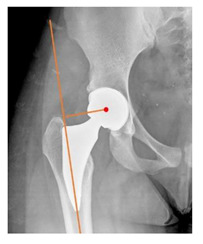
Canal fill ratio	The ratio between the width of the femoral component by the width of the intramedullary bone canal at 1 cm below the lesser trochanter [31]		
Stem osteointegration	Measured according to Engh [32] grading scale		
Acetabular osteointegration	Measured according to Moore [33] scale		
Heterotopic ossification	Classified according to Brooker [34] classification		

**Table 2:** Patient characteristics and baseline variables. All results are expressed as crude numbers for dichotomous variables and as mean ± Standard Deviation for continue variables.

*PATIENTS BASELINE VARIABLES*	
Patients (Male/Female)	68 (33/35)
Hips (Left/Right)	74 (44/30)
Mean Age at THR (years)	15.7 ± 1.7 (11–18)
Weight (Kg)	62.3 ± 14.9 (33–116)
Weight (z-score)	0.52 ± 0.34 (0–1)
Height (cm)	164.8 ± 10.8 (145–195)
Height (z-score)	0.44 ± 0.34 (0–1)
BMI (cm^2^/Kg)	22.8 ± 4.1 (14.7–33.3)
BMI (z-score)	0.63 ± 0.32 (0–1)
Follow-up (years)	6.6 ± 4.4 (2–20)

**Table 3:** Implants characteristics.

	N	%
**Acetabular cup’s size**		
42	12	16.2
44	6	8.1
46	16	21.6
48	13	17.6
50	11	14.9
52	6	8.1
54	5	6.8
56	1	1.4
58	4	5.4
**Cup’s commercial name**		
Fixa Ti-por^®^ (Adler)	44	59.5
EP-FIT PLUS™ (Smih & Nephew)	7	9.5
ALLOFIT^®^-S IT (Zimmer)	6	8.1
Other (less than 5 implanted)	17	23.0
**Stem’s commercial name**		
APTA (Adler-Ortho)	13	17.6
NANOS™ (Endoplant GmbH)	11	14.9
A-ACUTA S (Adler-Ortho)	7	9.5
Other (less than 5 implanted)	43	58.1
**Head material**		
Biolox^®^ or Biolox^®^Forte	14	18.9
BIOLOX^®^delta	60	81.1
**Head size**		
28	6	8.1
32	48	64.9
36	14	18.9
40	6	8.1
**Neck**		
Modular	27	36.5
Nonmodular	47	63.5

**Table 4:** Preoperative and postoperative radiographic parameters. All results are expressed as crude numbers for dichotomous variables and as mean ± Standard Deviation for continue variables. LCEA: Lateral center-edge angle. * Varus inclination is expressed as negative value, positive value for valgus.

*PREOPERATIVE Radiographic Parameters*	
LCEA	41.6 ± 1.1 (3–110.4)
Acetabular Index	9.1 ± 21.5 (−23.8–41)
Acetabular depth/width ratio	29.5 ± 10 (3.9–63.7)
Sharp Angle	39.2 ± 5.5 (20.1–51.2)
Neck-Shaft angle	135 ± 9.5 (103–157)
Risser+ (0–0+–1–2–3–4–5)	0%–0%–8%–2%–12%–46%–23%
***POSTOPERATIVE Radiographic Parameters***	
Cup Abduction	37.3 ± 8.8 (18.5–62.7)
Cup Anteversion	11.9 ± 5.7 (0–26)
Center of Rotation high	20.8 ± 5 (7.7–35.4)
Center of Rotation medialization	29.7 ± 4 (19.5–40.4)
Stem varus-valgus *	−1.2 ± 3.1 (−11–7.7)
Leg length discrepancy	−1.6 ± 7.9 (−27.9–16.3)
Femoral offset	39.2 ± 7.1 (27.6–53.2)
Canal Fill Ratio	75 ± 9.8% (50–95%)

## Data Availability

Data available on request due to restrictions. The data presented in this study could be available on request from the corresponding author. The data are not publicly available due to national privacy regulations.
